# Embolie de liquide amniotique: à propos de deux cas

**Published:** 2012-04-17

**Authors:** Houda Elbahraoui, Hanane Bouziane, Adil Elghanmi, Amina Lakhdar, Zaki Elhanchi, Driss Ferhati

**Affiliations:** 1 Service de gynecologie-obstetrique, CHU Ibn Sina Rabat, Maroc

**Keywords:** Embolie amniotique, hémorragie, arrêt cardiaque, foetus, Maroc

## Abstract

L’embolie de liquide amniotique (ELA) est une complication imprévisible de l’accouchement, souvent fatale, associant un collapsus cardiovasculaire sévère, un syndrome de détresse respiratoire aiguë et une hémorragie avec coagulation intra vasculaire disséminée (CIVD). Dès l’évocation du diagnostic, la prise en charge doit être multidisciplinaire et intensive. ELA est responsable d’une mortalité maternelle et néonatale importante, son incidence est extrêmement variable selon les études et le taux de mortalité maternelle varie entre 26 et 86 % selon les études. Ces dix dernières années, le pronostic materno-fœtal semble en amélioration grâce aux progrès de prise en charge standardisée multidisciplinaire sur les lieux d’accouchement. Nous rapportons deux cas d’embolie de liquide amniotique. Le premier cas s’est manifesté au cours du travail et le deuxième cas est survenu dans les suites immédiates de l’accouchement.

## Introduction

L’embolie de liquide amniotique (ELA) est une complication imprévisible mais grave du travail obstétrical. Elle correspond au passage de liquide amniotique dans la circulation maternelle, responsable d’un tableau associant des signes respiratoires, circulatoires, neurologiques et hématologiques. En l’absence de test simple, le diagnostic repose sur un faisceau d’arguments cliniques et sur l’élimination des diagnostics différentiels.

Sa survenue est imprévisible et brutale avec une détresse vitale pour la mère et le fœtus. La forme usuelle survient au cours du travail, souvent marquée par sa rapidité et après rupture des membranes [1].

La certitude diagnostique se fait par la mise en évidence à l’examen anatomopathologique de cellules amniotiques à différents niveaux: prélèvement d’une veine centrale, prélèvement de sécrétion broncho-alvéolaire, examen de la pièce d’hystérectomie ou l’autopsie. Cette certitude est difficile à obtenir. Ces examens ne sont pas toujours pathognomoniques car les squames fœtales et adultes sont très difficiles à différencier.[2]

## Patient et observation

### Cas 1

Madame A.M est une parturiente de 28 ans, troisième geste troisième pare et deux enfants vivants, sans antécédents médicaux ou chirurgicaux notables.la grossesse actuelle est non suivie estimée a 39 semaines d’aménorrhées, la patiente a été admise en salle de travail à 8 cm de dilatation cervicale avec une présentation céphalique fixé et une poche des eaux intacte. On a réalisé une rupture artificielle des membranes. La parturiente a présenté ensuite un malaise avec perte de connaissance et une crise convulsive généralisée ; sa tension artérielle baissait à 08 /04 mmHg avec apparition d’une bradycardie fœtale, une réanimation immédiate a était réalisé pour la mère, avec une extraction instrumentale rapide d’un fœtus apgar 3 /10 a la première minute passé à 6/10 après réanimation, pesant 3250g. Mais la patiente a présenté une hémorragie de la délivrance avec arrêt cardiorespiratoire, une délivrance artificielle a été réalisée au même temps que les mesures de réanimation avec perfusion d’ocytocique et introduction de 600µg de misoprostol en intra rectale ;l’activité cardiaque de la patiente a repris après 5 min de réanimation mais devant la persistance du saignement , l’atonie utérine et la coaculopathie de consommation (CIVD) ,une hystérectomie d’hémostase a été réalisée et une transfusion de 8 culot globulaire et 10 poches de plasma frais congelé. L’état hémodynamique s’est stabilisé avec arrêt du saignement.

La recherche d′éléments du liquide amniotique a été réalisée sur le sang maternel périphérique et sur une voie veineuse centrale confirmant la présence de cellules amniotiques compatible avec le diagnostic d’ELA.

Les suites ont été marquées par une normalisation du bilan biologique et une reprise de connaissance sans séquelles neurologiques pour la maman contrairement au nouveau-né qui avait des lésions neurologiques graves.

### Cas 2

Mme A.R est une parturiente de 40 ans ; cinquième geste troisième pare et deux enfants vivants. on retrouve dans ses antécédents deux accouchements par voie basse et un avortement spontané, la grossesse actuelle est bien suivie estimée a 40 semaines d’aménorrhée. la patiente a été admise pour accouchement: l’examen a son admission retrouvait un col effacé complètement et dilaté a 4 cm avec présentation de siège décomplété et une poche des eaux intact qu’on a respecté jusqu’à dilatation complète puis elle est rompue artificiellement suivie d’un accouchement podalique d’un nouveau-né de sexe masculin apgar 10/10 poids de naissance 3100g. La patiente a présenté ensuite une agitation avec dyspnée, cyanose, toux, douleurs thoraciques et une hypotension a 09/04 mmhg, elle a été prise en charge en urgence par l’équipe de réanimation, mais son état s’est compliquée par la survenue d’une hémorragie de la délivrance avec inertie utérine et arrêt cardiaque les mesures de réanimations se sont poursuivi ainsi qu’une perfusion d’ocytocique et l’utilisation de 800 µg de misoprostol en intra rectale. Après plusieurs minutes de massage, une activité cardiaque efficace a été retrouvée. Le bilan biologique était en faveur d’une CIVD, une hystérectomie d’hémostase a été réalisée au même temps qu’une transfusion de 10 culots globulaires et 12 poches de plasma frais congelé, mais le saignement a persisté avec des hémorragies diffuses tant dans le champ opératoire qu’aux points de ponction. Un nouvel arrêt cardiaque est survenu avant la fermeture de l’abdomen. La patiente est décédée une heure après malgré les mesures de réanimation. L’analyse histologique de la pièce d’hystérectomie a retrouvé une embolie veineuse de liquide amniotique.

## Discussion

L’ELA est une complication rare, survenant de façon brutale et responsable d’une mortalité maternelle et néonatale importante. Elle survient dans la majorité des cas pendant le travail et après la rupture des membranes (entre 70 et 90 % des cas) ou dans le post-partum immédiat (dans les cinq minutes). Mais des cas d’ELA ayant débuté plusieurs heures après l’accouchement ont été rapportées. [2]

La physiopathologie de l’ELA n’est pas clairement définie mais pourrait être due à un passage de liquide amniotique (LA) vers la circulation maternelle par rupture de la barrière utéro-placentaire. L’embole est constitué de squames épithéliales provenant de la peau de l’enfant, de cheveux, de vernix, de sécrétion intestinale (mucine) voire de méconium. Les emboles se logent dans les vaisseaux pulmonaires de moins d’un millimètre de diamètre et peuvent être retrouvés jusqu’à cinq semaines après l’accouchement [2–3].

Le tableau clinique brutal débute le plus souvent pendant le travail ou tout de suite après la naissance de l′enfant, par voie basse ou par césarienne. Des cas isolés sont rapportés en dehors de tout travail. [4]. Les principaux facteurs de risques sont l’hyperpression intra- utérine induite par un hydramnios, les amniocentèses, les amnio-drainages, les amnio-infusions, les tocographies internes, les morts fœtales intra-utérines et les traumatismes abdominaux. Des cas d’ELA ont été décrits à la suite d’interruptions médicales ou volontaires de grossesse, et même lors d’ablation de fil de cerclage. Ils sont cependant tous discutés. Les composants du liquide amniotique ont été incriminés dans la survenue d’une réaction allergique de type anaphylactique responsable d’une hypertension artérielle pulmonaire [2]. Les manifestations cliniques habituellement décrites dans l′ELA sont de deux types, hémorragiques et cardiovasculaires. Il est admis que la première conséquence physiopathologique est une hypertension artérielle pulmonaire (HTAP) sévère associée parfois à une atteinte du ventricule gauche (VG). L′HTAP relèverait de deux mécanismes, un phénomène mécanique pur avec obstruction plus ou moins complète des capillaires pulmonaires par du liquide amniotique, associé à une vasoconstriction pulmonaire secondaire à la présence en grande quantité dans le liquide amniotique d′endothéline, puissant vasoconstricteur, responsable d′une HTAP [5–6]. L′atteinte du VG est indirecte par compression par un ventricule droit dilaté ou directe par toxicité du liquide amniotique qui agirait sur la contractilité du myocarde et participerait au collapsus initial. L′arrêt circulatoire est fréquent et précoce, voire initial [7]. Une hémorragie de la délivrance favorisée par une CIVD due à la richesse du liquide amniotique en facteurs X activateur libérés dans la circulation maternelle [2].

La brutalité de survenue de l′ELA impose en priorité la mise en route immédiate de traitements symptomatiques qui excluent temporairement toute investigation paraclinique. Des examens biologiques permettent de guider la réanimation [4]. Le diagnostic biologique peut et doit être entrepris une fois les manœuvres de réanimation efficaces mises en route ou malheureusement post-mortem. La recherche d′éléments du LA (cellules épithéliales, vernix caseosa, lanugo, méconium) est réalisable sur le sang maternel périphérique ou sur une voie veineuse centrale, dans le liquide de lavage broncho-alvéolaire, et/ou enfin sur les tissus pulmonaires maternels en cas de décès de la patiente. Des réserves quant à la fiabilité diagnostique affectent chacun de ces examens. La présence de méconium dans le LA est observée à terme, ainsi que le lanugo et le vernix caseosa, mais si l′ELA survient plus précocement, ces éléments font habituellement défaut. Quant aux cellules de desquamation observées dans le sang maternel en cas d′ELA, leur origine fœtale serait pour certains auteurs discutables [4].

Le diagnostic d’ELA doit être évoqué devant toute manifestation cardiovasculaire et hémorragique survenant dans le péripartum. Il sera confirmé par des analyses anatomopathologiques. Il faut également penser aux autres diagnostics différentiels tel: l’infarctus du myocarde, l’embolie pulmonaire, l’embolie gazeuse, l’éclampsie, le choc septique ou anaphylactique [2].

En dehors du traitement symptomatique classique, peu de nouvelles thérapeutiques sont proposées. La prise en charge symptomatique initiale du collapsus n′a pas de spécificité, associant remplissage vasculaire et catécholamines. La réanimation d′un arrêt cardiaque chez une femme enceinte comporte quelques particularités et l′extraction fœtale précoce à deux objectifs: sauver l′enfant et améliorer la réanimation maternelle [4–8].

Les autres aspects de la prise en charge obstétricale pendant le travail de ces patientes incluant les techniques chirurgicales ou de radiologie interventionnelle n′ont rien de spécifique, hormis qu′elles finissent le plus souvent par l′hystérectomie d′hémostase de sauvetage maternel [4]. Au cours des dernières années, le pronostic de l′ELA semble modérément meilleur grâce à une meilleure prise en charge initiale multidisciplinaire. L′arrêt cardiorespiratoire est la cause principale du mauvais pronostic vital et des séquelles neurologiques maternelles. La présence d′un liquide amniotique méconial et la mort fœtale in utero sont aussi des facteurs de mauvais pronostic. Dans les cas d′évolution favorable, l′évolution ne présente pas de caractéristique spécifique. Enfin, la survenue d′une ELA ne contre-indique pas une grossesse ultérieure [4–9].

Le pronostic vital et neurologique du fœtus est corrélé au délai d′extraction chez les patientes les plus graves présentant un arrêt cardiorespiratoire [4].

Nous avons rapporté deux cas de patientes ayant présenté une ELA dont les évolutions ont été différentes. le diagnostic est d’abord clinique puis confirmé par l’analyse biologique et anatomopathologique.

Dans le premier cas, la patiente a présenté le tableau typique d’ELA au cours du travail mais grâce a une prise en charge rapide l’évolution a été marquée par une récupération totale de la patiente sans séquelles neurologiques ou cardiovasculaires.

Dans le deuxième cas la symptomatologie de l’ELA est survenue dans les suites immédiates de l’accouchement avec des complications graves et une évolution rapide vers le décès.

## Conclusion

Le diagnostic d’ELA doit être évoqué devant toute manifestation cardiovasculaire et hémorragique survenant dans le péripartum. Il sera confirmé par des analyses anatomopathologiques. Le pronostic peut être amélioré par: Une prise en charge diagnostique et thérapeutique rapide devant un arrêt cardiaque associé à une hémorragie de la délivrance d’apparition brutale et particulière par son caractère incoagulable; ? Une suspicion précoce du diagnostic d’ELA; un traitement agressif de la CIVD; l’élimination rapide des autres causes d’hémorragie de la délivrance; une réanimation efficace avec un transfert dans un service adapté.
